# A Comprehensive Genetic Analysis of Candidate Genes Regulating Response to *Trypanosoma congolense* Infection in Mice

**DOI:** 10.1371/journal.pntd.0000880

**Published:** 2010-11-09

**Authors:** Ian Goodhead, Alan Archibald, Peris Amwayi, Andy Brass, John Gibson, Neil Hall, Margaret A. Hughes, Moses Limo, Fuad Iraqi, Stephen J. Kemp, Harry A. Noyes

**Affiliations:** 1 Centre for Genomic Research, School of Biological Sciences, University of Liverpool, Liverpool, United Kingdom; 2 The Roslin Institute, University of Edinburgh, Roslin, United Kingdom; 3 International Livestock Research Institute, Nairobi, Kenya; 4 Faculty of Life Sciences, University of Manchester, Manchester, United Kingdom; 5 School of Computer Science, University of Manchester, Manchester, United Kingdom; 6 Egerton University, Njoro, Nakuru, Kenya; Yale School of Public Health, United States of America

## Abstract

**Background:**

African trypanosomes are protozoan parasites that cause “sleeping sickness” in humans and a similar disease in livestock. Trypanosomes also infect laboratory mice and three major quantitative trait loci (QTL) that regulate survival time after infection with *T. congolense* have been identified in two independent crosses between susceptible A/J and BALB/c mice, and the resistant C57BL/6. These were designated *Tir1*, *Tir*2 and *Tir3* for *Trypanosoma infection response*, and range in size from 0.9–12 cM.

**Principal Findings:**

Mapping loci regulating survival time after *T. congolense* infection in an additional cross revealed that susceptible C3H/HeJ mice have alleles that reduce survival time after infection at *Tir1* and *Tir3* QTL, but not at *Tir2*. Next-generation resequencing of a 6.2 Mbp region of mouse chromosome 17, which includes *Tir1*, identified 1,632 common single nucleotide polymorphisms (SNP) including a probably damaging non-synonymous SNP in *Pram1* (PML-RAR alpha-regulated adaptor molecule 1), which was the most plausible candidate QTL gene in *Tir1*. Genome-wide comparative genomic hybridisation identified 12 loci with copy number variants (CNV) that correlate with differential gene expression, including *Cd244* (natural killer cell receptor 2B4), which lies close to the peak of *Tir3c* and has gene expression that correlates with CNV and phenotype, making it a strong candidate QTL gene at this locus.

**Conclusions:**

By systematically combining next-generation DNA capture and sequencing, array-based comparative genomic hybridisation (aCGH), gene expression data and SNP annotation we have developed a strategy that can generate a short list of polymorphisms in candidate QTL genes that can be functionally tested.

## Author Summary

About one-third of cattle in sub-Saharan Africa are at risk of contracting “Nagana”—a disease caused by *Trypanosoma* parasites similar to those that cause human “Sleeping Sickness.” Laboratory mice can also be infected by trypanosomes, and different mouse breeds show varying levels of susceptibility to infection, similar to what is seen between different breeds of cattle. Survival time after infection is controlled by the underlying genetics of the mouse breed, and previous studies have localised three genomic regions that regulate this trait. These three “Quantitative Trait Loci” (QTL), which have been called *Tir1*, *Tir2* and *Tir3* (for *Trypanosoma Infection Response* 1–3) are well defined, but nevertheless still contain over one thousand genes, any number of which may be influencing survival. This study has aimed to identify the specific differences associated with genes that are controlling mouse survival after *T. congolense* infection. We have applied a series of analyses to existing datasets, and combined them with novel sequencing, and other genetic data to create short lists of genes that share polymorphisms across susceptible mouse breeds, including two promising “candidate genes”: *Pram1* at *Tir1* and *Cd244* at *Tir3*. These genes can now be tested to confirm their effect on response to trypanosome infection.

## Introduction

African trypanosomiasis is a disease of both livestock and humans, largely caused by three species of *Trypanosoma* parasites. Two subspecies of *T. brucei*: *T. b. gambiense* and *T. b. rhodesiense*, cause severe disease in humans, whilst disease in livestock is mainly caused by two other species: *T. vivax* and *T. congolense*. The diseases affect over ten million Km^2^ of Africa and it is estimated that some thirty percent of Africa's 160 million cattle are at risk of infection. Losses of livestock and crop production are estimated at over $1 billion per annum [1].

Some indigenous breeds of cattle, notably N'Dama (*Bos taurus*), have the ability to tolerate the effects of an infection by *Trypanosoma* parasites, and remain productive. Other, introduced, breeds are much more susceptible, and quickly show the classic symptoms of infection, such as anaemia, fatigue and muscle wastage [2]. This effect is under genetic control, and ten quantitative trait loci (QTL) have been mapped in F2 crosses between the N'Dama and susceptible Boran cattle (*Bos indicus*) [2].

Scientists are aided by a mouse model of trypanotolerance, as African trypanosomes also infect laboratory mice in which susceptibility is measured by survival time after infection, which varies between inbred lines. Whilst C57BL/6 mice survive for a relatively long period after infection with *T. congolense* (110 days), some other strains, such as A/J (16 days), 129/J (23 days), BALB/c (49 days) and C3H/HeJ (59 days) mice are relatively susceptible [3], [4], [5]. Mapping studies, initially undertaken in two independent F2 crosses: C57BL/6JOlaHSD (C57BL/6) × BALB/cOlaHsd (BALB/c) and C57BL/6JOlaHSD × A/JOlaHsd (A/J), identified three major QTL regulating survival time [6]. These were mapped to mouse chromosomes 17, 5 and 1 and have been designated *Tir1*, *Tir2* and *Tir3* respectively for *Trypanosoma Infection Response*. These loci were further refined to five smaller regions using advanced intercross lines of the same crosses that were extended to the F6 and then F12 generations, in which *Tir3* was resolved into three smaller regions, termed *Tir3a*, *Tir3b* and *Tir3c* [7], [8]. Whilst these studies substantially reduced the size of the 95% confidence interval of each of the QTL to between 0.9 and 12 cM, each one still includes 17 to 650 candidate genes.

Moving from well defined QTL regions to QTL genes is still a major challenge: over 2,750 such quantitative trait loci have been mapped in mice and rats but fewer than 1% have been characterised at the molecular level [9]. However, new sequencing technologies are making it possible to identify a large proportion of the differences between common inbred mouse strains. At present this is possible for defined areas of the genome, but public data sets will soon be available for the whole genome. We have used a combination of these methods and resources to demonstrate how large QTL regions can be reduced to tractable short lists of candidate genes for functional analysis.

We have mapped QTL in a C57BL/6 × C3H/HeJ cross so that we now know whether four mouse strains carry either the susceptible or the resistant allele at each QTL. This will reduce the number of polymorphisms that correlate with phenotype at any given QTL. The haplotype structure of the QTL regions has been determined using the 8 million public SNP from 16 mouse strains in the Perlegen set and identified regions where haplotypes correlate with survival time in the four mouse strains studied. Copy number variations (CNV) have been shown to be responsible for a significant number of quantitative traits [10]. We have used array comparative genomic hybridisation (aCGH) to identify CNV in QTL regions that correlate with survival in the four mouse strains. We have also correlated CNV with existing gene expression data from three of the mouse strains [11] to identify CNV that putatively cause expression differences. Finally we have sequenced one of the QTL regions in four strains of mice to identify SNP that correlate with phenotype and validated these against an additional publicly available dataset [12], [13]. We have also used Polyphen to identify the non-synonymous SNP in the QTL regions that are most likely to change the activity of the protein.

By combining additional mapping with haplotype analysis, aCGH and resequencing we have reduced the initial long list of genes within QTL regions to a short list of candidate genes with defined genetic differences that correlate with phenotype. It is now practical to test the function of these genes and polymorphisms to determine their role in response to infection with *T. congolense*. The Perlegen and aCGH data is already publically available for many mouse strains and the Wellcome Trust Sanger Institute is resequencing the genomes of the common laboratory mouse strains so this strategy will soon be applicable to many QTL without further experimental work [13], [14], [15].

## Methods

### Ethics statement

All animal work was undertaken under IACUC ref no 2003.19. The ILRI IACUC complies voluntarily with the UK Animals (Scientific Procedures) Act 1986 that contains guidelines and codes of practice for the housing and care of animals used in scientific procedures. All animals on survival experiments were regularly monitored to check for signs of terminal illness, and any showing such signs were euthanised by UK Schedule 1 procedures.

### C3H/HeJ × C57BL/6 cross

C57BL/6JOlaHSD (C57BL/6) and C3H/HeJ mice were obtained from Harlan Laboratories. Mice were infected with 4×10^4^ *T. congolense* strain IL1180 intra-peritoneally (ip) as previously described [6]. Any mice that did not develop a microscopically proven parasitaemia were removed from the study.

345 F2 C3H/HeJ × C57BL/6 mice were phenotyped for survival time after infection with *T. congolense* strain IL1180. 94 animals that had extreme survival times (≤62 days and >140 days) were selected for genotyping using the markers shown in Table S1 in Supporting Text S1. Selective genotyping significantly reduces genotyping costs with little loss of power to detect QTL, however it does give exaggerated estimates of effect sizes [16]. The F2 mice were also genotyped at the *Tlr4* locus since C3H/HeJ carries a proline to histidine mutation at position 712 of the *Tlr4* gene that makes this mouse strain insensitive to LPS and might modify response to infection with *T. congolense* [17].

PCR reactions were performed using Reddymix (Thermo) with 20 ng of template DNA. Cycling conditions were as follows: 95°C, 50 secs; [Tm −5]°C, 50 secs; 65°C, 50 secs; 30× cycles. PCR products, including negative controls, were resolved by ethidium bromide stained agarose-gel electrophoresis and visualised under UV-light. SNP were genotyped by sequencing PCR products using primers shown in Table S2 in Supporting Text S1. Unincorporated primers and residual nucleotides were degraded using ExoSAP-IT (USB Corp, Ohio, USA) and sequencing products generated using Big-Dye v3.1 terminators (Applied Biosystems, Foster City, USA). Cycle sequencing products were ethanol precipitated and subject to electrophoresis on an Applied Biosystems ABI-3130XL capillary sequencer. Microsatellite and SNP genotyping data was viewed using PeakScanner (Applied Biosystems) and GAP4 [18] respectively.

### Allocation of strains to haplotypes

Strains were allocated to haplotypes as previously described [19], [20]. Briefly, Perlegen SNP and haplotype boundaries were downloaded from Perlegen [15]. Strains were allocated to haplotypes for each haplotype block using a local Perl script that extracted all alleles from the Perlegen dataset within a haplotype block, substituted them into the C57BL/6 reference sequence and submitted the resulting aligned sequences to the Jukes-Cantor algorithm in DNADIST in PHYLIP to calculate genetic distances between each pair of strains [21]. Strains were given a binary “barcode” with all possible pairs of strains assigned a 1 or a 0 depending on whether the genetic distance for that pair was above or below a threshold value. Strains that had the same “barcode” were allocated to the same haplotype number. C57BL/6 was used as the reference strain for block allocation and assigned to haplotype one; Succeeding strains were allocated to the same haplotype block as another strain that they shared a haplotype with or, if there was none, to the next available haplotype number (Full details are available in Supporting Text S1; Allocation of strains to haplotypes).

### CNV discovery

Array CGH was performed using the Agilent Mouse Genome CGH Microarray 244A platform. Dye-flip replicates were carried out on the C57BL/6 reference strain and three test strains (129P3/J, A/J and BALB/cJ) and analysed as previously described [22]. Overlapping aberrations were grouped into CNVR (t-test analysis, P≤0.05, Overlap 0.9) using the Agilent CGH analytics software (v 4.0) and using the ADM-2 algorithm (threshold 6.0) using centralization (threshold 6.0, bin size 1) and Fuzzy Zero [23]. CGH array data have been submitted to the NCBI Gene Expression Omnibus database (GEO) [GEO: GSE9669].

### DNA capture and sequencing

Genomic DNA for BALB/cJ (Jackson #000651), 129P3/J (Jackson #000690), A/J (Jackson #000646) and C3H/HeJ (Jackson #000659) were obtained from the Jackson Laboratories and submitted to Nimblegen for sequence capture [24]. Capture probes were designed to cover 4.5 Mbp of non-repetitive sequence between 30,637,692 bp and 36,837,814 bp on Mmu17 (NCBI37). 385,000 60mer probes were tiled at approximately 5 bp intervals leading to a mean of 12 probes over each base. Captured DNA was sequenced on a Roche 454 FLX Genome Sequencer using Titanium chemistry (Roche). Sequence assembly and SNP calling was performed using the Newbler mapping algorithm, which aligned 454 reads against the Ensembl C57BL/6 reference (NCBI37) and outputs lists of SNP and associated coverage metrics.

As pyrosequencing is known to miscall sequences either across, or either side of, homopolymeric tracts (long stretches of a single nucleotide), discrepancies were removed from subsequent analysis if they were within 13 bp of a homopolymeric tract ≥5 bp [25]. SNP were additionally filtered to those with at least an eight-fold coverage and occurring in at least 87.5% of the reads sequenced across any polymorphic position. 14,440 high-confidence genotypes were submitted to dbSNP with SSIDs ss159831440-ss159845897. 454 reads were submitted to the European Short Read Archive under Accession number ERA000179.

SNP were aligned against coding sequences and non-synonymous SNP were identified. SNP positions were compared to the mouse regulatory build to test for SNP that may alter transcription factor binding sites or promoter regions [26], [27]. A 24-bp insertion in *Mdc1* in susceptible strains was amplified by PCR and verified by agarose gel electrophoresis, but could not be shown to have any functional effect (data not shown).

### Identification and annotation of single nucleotide polymorphisms (SNP)

SNP outside the *Tir1* region were obtained from the 8 million Perlegen SNP set [15]. phastCons conservation scores for SNP positions [28] were obtained from UCSC [29]. These scores are a measure of how conserved a position is amongst 30 mammalian species and are on a scale between 0–1 with the most conserved positions scored as 1.

SNP within exons were annotated using the Ensembl SNP annotation API to identify non-synonymous SNP (nsSNP) and SNP in splice sites. nsSNP in the 454 data were identified with a local Perl script. Publically available functional SNP were also obtained from BioMart and the Wellcome Trust Sanger Institute website [12].

nsSNP were annotated with Polyphen [30] using the Polyphen batch submission tool. Publicly available functional SNP identified at QTL for which complete genotypes were not available were confirmed in C57BL/6, A/J, BALB/cJ and 129P3 mice using PCR and dideoxynucleotide sequencing as described for genotyping. Sequences which showed evidence of multiple copies were cloned using TOPO-TA cloning kit (Invitrogen) and sequenced.

### Measurement of gene expression

Gene expression was measured for A/J, BALB/c and C57BL/6 mice before infection and at four time points post infection on Affymetrix 450_2 microarrays as previously described [11]. All microarray data has been deposited at ArrayExpress under the accession number E-MEXP-1190. The expression data and plots like those presented here are also available for all genes on the microarrays from the authors' website [31].

## Results

### Refining numbers of candidate genes within the *Tir* QTL

#### Determination of QTL boundaries and initial candidate gene identification

Different locations of the *Tir2* and *Tir3a,b,c* QTL have been published at the F6 and F12 generations [7], [8]. QTL have also been physically mapped using congenic mice [32]. The congenic data supports the F6 location in one case (*Tir2*) and the F12 location in one other (*Tir3a*). Consequently we have annotated genes under both definitions of QTL positions and discuss their relative merits, case by case, below. In order to refine the number of candidate genes within *Tir* QTL it is necessary to first convert the 95% confidence intervals of the QTL from centiMorgan (cM) positions to megabase (Mbp) positions. Whilst the exact assignment of physical boundaries to the QTL is not possible, we have used the physical position of the peak marker in the F6 and F12 advanced intercross studies [7], [8] as the most likely position of the peak of the QTL. We estimated the physical size of the 95% confidence interval (CI) by using Mouse Genome Informatics data to find the median Kbp/cM ratio for the intervals between the ten flanking markers (which were spaced at ∼0.3 Mbp intervals). This ratio varied between 0.69–5.43 Mpb/cM and was used to convert the 95% CI in cM to Kbp. These positions are then used to identify the candidate genes contained within the QTL prior to further refinement (Table 1).

**Table 1:** Physical locations of QTL and counts of candidate genes.

QTL	*Tir1*	*Tir2*-F6	*Tir2*-F12b	*Tir3a*-F6	*Tir3a*-F12	*Tir3b*-F6	*Tir3b*-F12	*Tir3c*-F6	*Tir3c*-F12
**Chromosome**	17	5	5	1	1	1	1	1	1
**Peak marker**	D17Mit16	D5Mit114	D5Mit58	D1Nds2a	DiMit286	D1Mit102	D1Mit102-DiMit105	D1Mit113	D1Mit107-DiMit16
**95%CI (cM)**	0.9	12	1	1.8	6	10	7	8	2
**Median Mbp per cM**	1.04	1.77	1.46	3.9	1.93	5.49	1.92	0.69	1.16
**Start (Mbp)**	33.27	71.02	73.45	100.54	124.71	121.63	148.15	170.96	164.3
**End (Mbp)**	34.2	92.3	73.91	107.57	136.19	176.56	161.44	176.51	166.6
**Size (Mbp)**	0.93	21.25	1.46	7.03	11.56	54.93	13.44	5.54	2.23
**# Genes**	43	210	27	20	127	650	113	143	35
**Number of Candidate Genes (H1)**	0	42	12	10	33	144	30	54	8
**Number of Candidate Genes (H2)**	27	74	14	10	63	355	61	122	8
**454 Sequencing Data**									
**Common SNP (d)**	194								
**Common nsSNP**	2								
**Additional Data from Illumina Comparison ** [**13**]									
**nsSNP**	0								
**5′-UTR SNP**	0								
**Synonymous SNP**	2								

Positions were interpolated using NCBI37 from peak marker positions and 95% confidence intervals. The physical position of the D1Nds2 marker is not known, so its position was estimated from the intervals between its flanking markers. Lists of the genes with different haplotypes are shown in the Supplementary Data S2: *GenesAndHaplotypes.xls*. ^a^ Number of SNP common to the three susceptible strains of mice: A/J; BALBc/J and C3H/HeJ. ^b^At Tir2-F12 we have estimated the physical 95% confidence interval around the D5MIT58 peak marker and this 1.46 Mb region contained 27 genes, however the exact position of the peak is hard to identify since both D5MIT58 and DMIT258 are at 41 cM in the MGI map although they are 7 Mb apart on the physical map.

Numbers of candidate genes were calculated under two hypotheses: Hypothesis 1: all four susceptible strains have the same haplotype as each other and different from C57BL/6. Hypothesis 2: All susceptible strains have a different haplotype from C57BL/6 but not necessarily the same as each other. Hypothesis 1 is a special case of hypothesis 2 and all genes included under hypothesis 1 are also included under hypothesis 2. Only A/J and BALB/c are known to carry susceptibility alleles at *Tir2* and so at this locus only the correlation of C57BL/6, A/J and BALB/c was considered. ^e^ nsSNP loci submitted to dbSNP.

### Identification of QTL in C3H/HeJ mice

By increasing the number of breeds known to carry susceptible alleles at the QTL, candidate gene lists can be refined to remove those genes that are in QTL for *T. congolense* infection response but have the same ancestral haplotype as the resistant strain in at least one susceptible mouse breed. The three major *Tir* QTL have only been identified in C57BL/6, A/J and BALB/c mice, with C57BL/6 carrying the resistant allele at each locus. To that end, we measured survival after infection in an inter-cross between another susceptible breed, C3H/HeJ, and C57BL/6 mice. For the cross, the mean survival times of parental founder lines for the C3H/HeJ × C57BL/6 F2 cross were 63 days for C3H/HeJ and 87 days for C57BL/6. Out of the 345 F2 C3H/HeJ x C57BL/6 mice that were phenotyped, we selectively genotyped the 94 mice (51♂ and 43♀; p = 0.41) that had the most extreme survival times (Table S1 in Supporting Text S1) with microsatellite and SNP markers across the three known QTL. Table 2 shows that C3H/HeJ carries alleles that reduce survival time at the *Tir3* QTL on Mmu1 and the *Tir1* QTL on Mmu17. No QTL was discovered on Mmu5 in the region of *Tir2*.

**Table 2:** Loci regulating survival after *T. congolense* infection in the C3H/HeJ × C57BL/6 cross.

Chr	F-value	LOD score	95% CI (cM)	QTL position (cM)	QTL effect days	Peak marker
17	17.22	6.344	17	16	32	D17mit81
1	9.13	3.614	47	94.9	24	D1mit356

94 mice were genotyped with markers across known QTL regions but not elsewhere in the genome. QTL effects are the mean number of days difference in survival between mice that are homozygous for the alternate alleles at a QTL. Positive QTL effects indicate that longer survival was associated with C57BL/6 alleles. The QTL effects are likely to be biased upwards as a consequence of selective genotyping of the extremes of the phenotypic distribution [16]. Phenotype distribution is shown in Figure S1 in Supporting Text S1.

### Refining numbers of candidate genes by allocation of alleles to haplotype blocks

Over eight million SNP and haplotype block boundaries derived from them have been published for the whole mouse genome [15], however the haplotype alleles carried by each strain are not available on a genome-wide basis. Full details of the allocation of strains to haplotype blocks and associated figures are available in Supporting Text S1; Allocation of Strains to Haplotypes. Results are briefly presented here:

In order to identify haplotype alleles that correlated with phenotype we obtained the Jukes-Cantor distance between each pair of the four mouse strains (C57BL/6, A/J, BALB/c and C3H/HeJ) for each haplotype block across each QTL. The distribution of the natural logarithm of Jukes-Cantor distances was approximately normal and the fifth percentile of the distribution corresponding to a distance of 5×10^−5^ was selected as a threshold (Figure S2 in Supporting Text S1). Strains were allocated to the same haplotype allele if the Jukes-Cantor distance between them at that block was less than 5×10^−5^.

Under the assumption that where QTL coincide in multiple crosses, it is likely that it is due to the same polymorphism in all breeds tested, there are only two possible distributions of resistant and susceptible haplotypes. In the present case, C57BL/6 was the only strain carrying a haplotype for longer survival at any QTL, therefore either: all susceptible breeds have the same haplotype that is different from C57BL/6 (hypothesis one); or C57BL/6 has a unique haplotype that differs from all susceptible breeds but that these might differ amongst themselves (hypothesis two).

A list was compiled of the genes for which the susceptible strains were on a different haplotype or immediately upstream or downstream of a different haplotype from the resistant (C57BL/6) strain (Supplementary Data S2: *GenesAndHaplotypes.xls*). Table 1 shows the number of candidate genes in each locus under the two nested hypotheses: H1) Short survival time is caused by a common deleterious allele in all three susceptible strains; or H2) that the difference in survival is attributable to a beneficial allele in the single long surviving line (C57BL/6) and the susceptible lines may carry any non C57BL/6 haplotype. Under H1 the number of candidate genes was reduced from 1193 to 283 and under H2 the number was reduced from 1193 to 651.

### A null allele of the *Tlr4* gene in C3H/HeJ does not affect survival

A functional toll like receptor 4 (*Tlr4*) gene is necessary for maximal control of *Trypanosoma cruzi* in mice [33] and there is evidence that the GPI anchor of *T. brucei* VSG has endotoxin like properties that could stimulate *Tlr4* [34]. C3H/HeJ has a polymorphism in the *Tlr4* gene, on mouse chromosome four, which ablates its function, making these mice insensitive to LPS [17]. We used this spontaneous mutation to discover whether *Tlr4* was as important in the response to *T. congolense* as to *T. cruzi*. Since all previous mapping had been done in mice with intact *Tlr4* genes, no QTL could have been detected at this locus even if *Tlr4* does modulate the response to infection. The C3H/HeJ × C57BL/6 mapping population could therefore be used to discover whether this gene (or a closely linked one) is involved in the regulation of survival time after infection. Mice were genotyped with a microsatellite marker linked to the functional polymorphism and sequenced across the polymorphic position. There was no association with either of these markers and survival time, indicating that the *Tlr4* pathway does not affect survival after *T. congolense* infection in mice (Table S1 in Supporting Text S1).

### Comparative genomic hybridisation and gene expression

To assess the impact of copy number variation regions (CNVR) upon the expression of genes that may influence response to *T. congolense* infection we performed array-based comparative genomic hybridisation (aCGH) on the complete genome of three mouse strains: 129P3, A/J and BALB/c, relative to C57BL/6. The expression of genes within CNVR in A/J, BALB/c and C57BL/6 mice over the course of infection was evaluated using a previously described dataset [11].

Genome-wide, one hundred and twenty-nine CNVR involving three or more probes were common to A/J, BALBc/J and 129P3/J. These encompassed a total of 317 genes, and ranged in size from 400 bp to 6.4 Mbp, although 96% were smaller than 1 Mbp. Twelve CNVR containing the complete coding sequences of genes and that had corresponding differences in gene expression, were common in all susceptible breeds of mice tested. Lists of the genome-wide CNVR is shown in Table S8 in Supporting Text S1.

**Figure 1:**
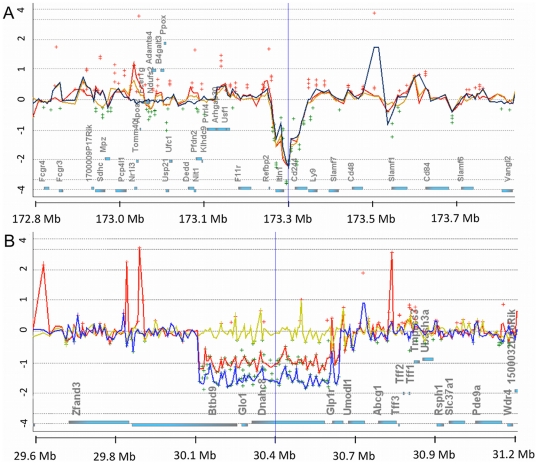
CNV plots from Agilent DNA Analytics software. A: Reduced copy numbers in C57BL/6 of *Itlnb* and *Cd244* near *Tir3c* relative to two susceptible breeds of mice (Chr 1: 172,831,532–173,931,532 bp). B: CNV data at the proximal end of *Tir1* showing a deletion of *Glo1* and *Dnahc8* in C57BL/6 and BALB/c relative to A/J and 129P3. (Chr 17: 29,854,972–30,954,972). Probes are plotted at their genomic position relative to their respective log_2_ fluorescence intensity ratios (Y-axis) along with genes on the x-axis (filled blue rectangles). Green dots are negative ratios and red dots positive ratios (threshold 0.5). Lines are a moving average over a 10 Kbp window for A/J (blue); 129P3 (red) and BALBc (yellow). Genomic positions are based on mouse build mm8 (NCBI36).

**Figure 2:**
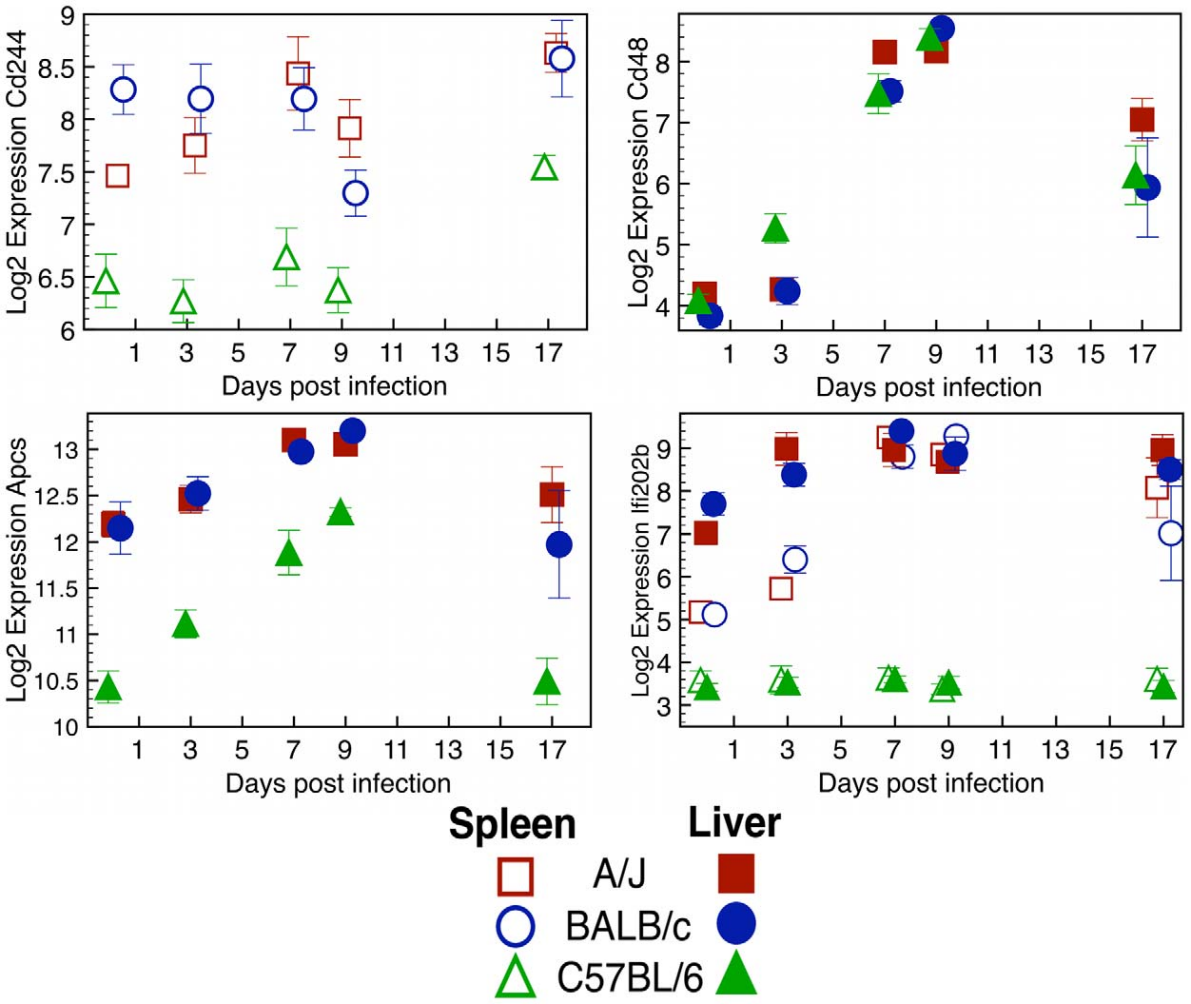
Expression of A/J OlaHsdnd (A/J), BALB/cJ OlaHsdce (BALB/c) and C57BL/6JOlaHSD (C57BL/6) mouse genes in the *Tir3c* locus at five time points in the course of infection (0 days; 3 days; 5days; 9 days; 17 days). Graphs include a small x-axis offset to improve spatial clarity. **A** *Cd244* in the spleen, **B** *Cd48* in the liver, **C** *Apcs* in the liver **D** *Ifi202b* in liver and spleen. *Cd244* expression was low in liver in all strains until Day 7 when it rose above background and C57BL/6 had slightly lower levels than A/J or BALB/c (data not shown).

One significant CNVR was detected close to the peak of *Tir3c* in the F6 population (D1Mit113: 173,734,611 bp). A two to four-fold reduction in C57BL/6 copy number relative to A/J, BALB/c and 129P3/J encompassed, or overlapped with, the coding sequences of *Itln1* (intelectin 1), *Cd244*, and *AC083892.19-1* and may affect the nearby *Ly9* (lymphocyte antigen 9) (173,441,746-173,499,029 bp; 11 probes; p = 0.0003; Figure 1A). There were expression differences in *Cd244* (Figure 2A), but not *Itln1* or *Ly9* [31], over the course of infection between resistant C57BL/6 and susceptible A/J and BALB/c. *AC083892.19-1* was not on the expression microarray. This CNV region has also been previously reported by Graubert *et al* [14] who showed that an additional susceptible strain, C3H/HeJ, carries the same variant as A/J and BALB/c.

No common CNVR were detected within *Tir1* or *Tir2*. The CNVR that was previously reported to be the cause of differential expression of Glyoxalase 1 (*Glo1*) [35] and is 2.8 Mbp from the peak of *Tir1*, was detected as a two to fourfold reduction in copy number for C57BL/6 and BALB/c relative to A/J and 129P3 (Chr17: 30,176,153 bp–30,650,413 bp; 68 probes; p<0.001; Figure 1B). Since the CNVR did not correlate with phenotype, this polymorphism is unlikely to contribute to the difference in response to infection.

### Identification of functional SNP

Lists of published non-synonymous SNP (nsSNP), SNP in splice sites; and regulatory regions and SNP that cause gain or loss of stop codons were obtained from BioMart. nsSNP were annotated using Polyphen [30] in order to identify those most likely to modify gene function. A complete list of annotated SNP is in Supplementary Data S1: *AnnotatedFunctionalSNP.xls*. Polyphen classifies nsSNP as benign, possibly damaging or probably damaging according to the likelihood that the polymorphism will modify protein activity. ‘Damaging’ implies a change of activity or function but this change could be beneficial to the animal.

#### *Tir1*

The physical size of the 95% CI for *Tir1* based on the combined data from the A/J × C57BL/6 and BALB/c × C57BL/6 F6 crosses [7] was 930 Kbp and contained 43 genes. *Tir1* was not reassessed with the F12 data. Assessing the Perlegen dataset against the smallest *Tir1* definition, none of the genes had haplotypes that correlated with phenotype under hypothesis 1, but there were 27 genes that correlated with phenotype under hypothesis 2 (Supplementary Data S2: *GenesAndHaplotypes.xls*). SNP that might modify phenotype at *Tir1* are discussed under sequencing of *Tir1* below.

#### *Tir2*

The *Tir2* QTL contained 210 genes in the 21.25 Mb (F6) QTL or 27 genes in the 1.46 Mb (F12) region, which was a subset of the F6 region. Congenic mice that were bred to physically map the *Tir2* QTL had a region of C57BL/6 DNA in an A/J background between 75.1 Mb and 89.7 Mb on chromosome 5 [32]. This was within the large F6 QTL (71.0–92.3 Mbp) but distal to the much smaller F12 QTL (73.5–73.9 Mbp). Since the QTL was physically mapped in the congenic mice, they are expected to provide a more accurate prediction of location than genetic mapping methods. There were 21 and 52 genes consistent with hypotheses 1 and 2 respectively within the congenic region (Supplementary Data S2: *GenesAndHaplotypes.xls*). There were probably damaging nsSNP in *Srp72* (signal recognition particle 72 kDa) and *Ugt2b38* (UDP glucuronosyltransferase 2 family, polypeptide B38) (Supplementary Data S1: *AnnotatedFunctionalSNP*). The SNP in *Ugt2b38* and *Srp72* had phastCons scores of <0.1 and 0.998 respectively indicating that the *Srp72* was in a highly conserved position. Therefore the *Srp72* SNP was the SNP with the greatest probability of having an effect on gene function in the *Tir2* congenic region, although what this might be and whether it would modify response to *T. congolense* is not known.

#### *Tir3a*

The F6 *Tir3a* locus, at around 103 Mbp on chromosome 1, is within a region that was tested for its effect on survival after *T. congolense* infection by breeding congenic mice that had a fragment of C57BL/6 origin between 93–123 Mbp on an A/J background [32]. There was no difference in survival between mice that carried the region derived from C57BL/6 and littermate controls without the C57BL/6 region indicating that the F6 region was not likely to contain the QTL gene. The F12 *Tir3a* locus was distal to the congenic region and is consequently a more likely candidate region for this QTL than the F6 QTL. It contains 33 and 63 candidate genes under hypotheses 1 and 2 respectively. These include *IL10, Cd55* (complement decay-accelerating factor) and *Cxcr4* (CXC chemokine receptor 4), which all have plausible roles in the response to infection but there were no published SNP in exons of any of these and no SNP in conserved intergenic regions. *Thsd7b* (Thrombospondin type-1 domain-containing protein 7B Precursor) was the only gene in the region with a probably damaging (Polyphen) SNP and this SNP was also in an evolutionary conserved position. However there are no published studies of *Thsd7b* and expression levels are low in all tissues measured [36].

#### *Tir3b*

The *Tir3b* region was the largest QTL in the F6 (54.9 Mb) and F12 (13.4 Mb) and contains 650 and 113 genes respectively, of which 144 and 30 have haplotypes that correlate with phenotype. The F6 *Tir3b* QTL overlaps the *Tir3a* and *Tir3c* loci but exclusively contained *Ptprc* (protein tyrosine phosphatase, receptor type, C; Leukocyte common antigen Precursor, CD45 antigen), which had a probably damaging nsSNP in a highly conserved position (phastCons score 1). *Tir3b* F12 and F6 both contained *Soat1* (Sterol O-acyltransferase 1), which had a probably damaging SNP. *Soat1* expression increases eight-fold after infection with *T. congolense* in A/J, BALB/c and C57BL/6 [31], and expression was up to four-fold higher in C57BL/6. *Soat1* is clearly responding to infection and the probably damaging SNP could affect its function and may be contributing to the difference in expression.

#### *Tir3c*

There were 122 and 8 genes at the F6 and F12 *Tir3c* loci that had haplotypes that correlate with phenotype, 54 and 8 of which had identical haplotypes in all the susceptible strains. *Cd244* (natural killer receptor 2B4) has a haplotype and expression pattern that correlates with phenotype (Figure 2A), as well as a CNV that may be the cause of the observed expression differences. It is a strong candidate for being a QTL gene at *Tir3c*. CD244 binds CD48 on lymphocytes and *Cd244* is about 60 Kbp from *Cd48*, which has a probably damaging nsSNP (rs31533394).

Additional candidate genes at *Tir3c* were *Apcs* (serum amyloid P-component; *Sap*) and *Ifi202b* (interferon activated gene 202B). The expression of *Apcs*, a major acute phase protein, rose after infection in all strains, but was consistently lower in C57BL/6 (Figure 2C). This was associated with a SNP (rs47990301) in a regulatory region that correlated with expression and phenotype and a SNP in a splice site in the 5′-UTR (rs47985673). Likewise, expression of *Ifi202b* increased to high levels after infection in A/J and BALB/c but remained at the threshold of detection in C57BL/6 in both liver and spleen. The *Ifi200* cluster, which includes *Ifi202b*, is at the distal end of *Tir3c* and contains genes that are all IFN-inducible and contain a highly conserved 200 amino acid motif [37]. *Fcgr3*, a low affinity immunoglobulin receptor that is associated with chronic inflammation [38], had a probably damaging nsSNP that correlated with phenotype. *Arhgap30* a little known rho-GTPase that is most highly expressed in macrophages and monocytes, had a probably damaging SNP (rs31539487) that correlated with phenotype in all strains tested. Similarly, we confirmed nsSNP in *Klhdc9* (rs45643169); *Darc* (Duffy blood group, chemokine receptor; rs51259593); *Slamf8* (signalling lymphocytic activation molecule F8; rs50073880) and *E430029J22Rik* (ENSMUSSNP3208701) that correlated with phenotype within this QTL region.

### Sequence capture and sequencing of *Tir1*

DNA from across the *Tir1* QTL was sequenced in order to characterise novel SNP and to improve the identification of alternate alleles for each haplotype block. DNA from four mouse breeds: 129P3, A/J, BALB/c and C3H/HeJ; was captured on Nimblegen arrays with probes for a 6.2 Mbp region of mouse chromosome 17 between 30,637,692 and 36,837,814 (NCBI37). 1.7 Mbp of repetitive sequence was excluded. Captured DNA was sequenced on a Roche 454 Genome Sequencer FLX using Titanium chemistry. 1,308,175 reads were mapped to the C57BL/6 reference sequence giving an average ∼15× coverage of each sequenced strain (mean read length 282 bp; total sequence ∼370 Mbp).

As 454 pyrosequencing is known to suffer from sequencing errors within, or close to long homopolymeric tracts, SNP were filtered to exclude those that were within a 13 bp window of homopolymeric tracts ≥5 bp. Furthermore, SNP were additionally filtered for those that were not outside regions covered by capture probes even if they were within the *Tir1* region. After filtering, 14,440 SNP loci were identified, 3,618 of which were not in dbSNP build 128. 1,588 loci were common to A/J, BALB/c and C3H/HeJ, but differed from C57BL/6. Furthermore, upon adding data for 129P3, there were 466 SNP loci common to all four sequenced mouse strains. Summary statistics for all SNP are available in Table S4 in Supporting Text S1.

**Figure 3:**
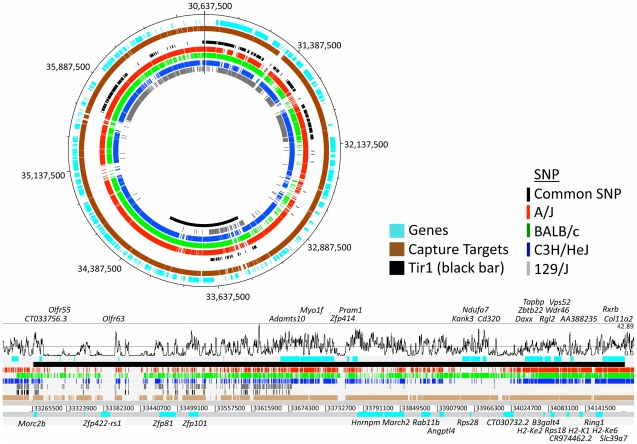
Array-based sequence capture and next generation sequencing of a 6.2 Mbp region of Mmu17 in four breeds of mice: A/J; BALB/c; C3H/HeJ and 129/J (Mmu17:30,637,692–36,837,814 bp). Plot is circular for ease of display [56]. *Tir1* is highlighted in black on the inside track. Genomic positions are in Mbp. The outer tracks (blue and brown) show genes and designed capture probes, respectively. The four, coloured, inner tracks show SNP called in each of the four sequencing experiments, with the black tick marks highlighting areas of common SNP. Haplotype blocks can clearly be seen as clustering of high- and low- density regions of SNP. A magnified region around *Tir1* is displayed underneath the circular plot. Tracks are identically coloured and include a moving average (window 1 Kbp) of sequence read coverage across the region (top). Genes in the region are displayed for the forward strand (above) and reverse strand (below).

Figure 3 shows a circular plot of all SNP called by the Roche/454 mapping algorithm (Newbler) against the C57BL/6 reference. Haplotype blocks can be seen as clusters of high-densities and low-densities of SNP. Whilst at this resolution it is not easy to see haplotype blocks in the A/J, BALB/c or C3H/HeJ data, one haplotype block stands out in the 129P3 data where 81 common SNP clustered within a 430 Kbp region (33,245,853–33,675,688 bp).

In order to validate SNP calls, 454-generated SNP were compared against those called in a recently published set sequenced on the Solexa/Illumina platform from flow-sorted mouse chromosome 17 for A/J [13], and similar, publicly available SNP from the concurrent Mouse Genomes Project (Wellcome Trust Sanger Institute) for BALB/c, C3H/HeJ and 129P2 mouse breeds [12]. Only 3 out of 36,784 (0.014%) of the homozygous calls (coverage >1; alternative allele frequency (AAF) >80%) were discordant between the two datasets Table S7 in Supporting Text S1. The 454 data included 53–71% of SNP in the Illumina data depending on the coverage required to call a SNP and the Illumina data contained 94–97% of SNP in the 454 data (Figure S5 in Supporting Text S1). Full details of the comparison are available in Supplementary Data S3; SNP validation.xls.

### Structural polymorphisms

Using all available data, the *Tir1* region contained 80 nsSNP loci that correlated with phenotype. There were seven “possibly damaging” (Polyphen) nsSNP and “probably damaging” nsSNP in PML-retinoic acid receptor alpha regulated adaptor molecule 1 (*Pram1*) (rs33399614), *Rgl2* (Ral guanine nucleotide dissociation stimulator-like 2) and *CR974462* (Table 3). Nine genes contained splice site polymorphisms (See Supplementary Data S1: *AnnotatedFunctionalSNP.xls*).

**Table 3:** nsSNP loci within the extended *Tir1* definition.

Position	C57BL/6	A/J	BALB/c	C3H/HeJ	Phast Cons	Gene	Polyphen Consequence	Peptide shift
33,283,941	A		G	G	<0.1	*Zfp421*	possibly damaging	Y/C
33,781,645	T	C	C	C	<0.1	*Pram1*	probably damaging	L/P
33,956,791	T		C		<0.1	*Kank3*	possibly damaging	S/P
34,069,285	C	T	T	T	0.928	*Rgl2*	probably damaging	H/Y
34,112,420	T	C	C	C	<0.1	CR974462.5	probably damaging	H/R
34,114,833	C	–	–		<0.1	CR974462.5	possibly damaging	G/R
34,119,278	G	A			<0.1	AA388235	possibly damaging	R/H
34,119,383	G	A			<0.1	AA388235	possibly damaging	G/D
34,119,473	T	C			0.337	AA388235	possibly damaging	F/S
34,134,481	T	C		C	<0.1	H2-K1	possibly damaging	H/R

Genes within *Tir1* (Mmu17:33271855–34203529 bp) with damaging nsSNP that correlate with survival phenotype. A full list of annotated SNP is available in Supplementary Data S1: *AnnotatedFunctionalSNP.xls*.

### Regulatory polymorphisms

Differences between the susceptible strains and C57BL/6 were aligned to the Ensembl mouse regulatory build (NCBI37: Ensembl 54). Ten differences were predicted to fall within regions of accessible chromatin and may affect transcription factor binding regions. Furthermore, 13 differences mapped to within 2500 bp of the upstream region of genes that may be associated with promoter regions. In total, 14 genes may be affected by SNP in this way (Table S5 in Supporting Text S1). Of the 13 genes for which microarray data was available, however, only phosphodiesterase 9A (*Pde9a*) showed any differences in gene expression, and these correlated with alleles of a SNP (rs33223038). A/J differed from C57BL/6 and BALB/c at this locus in both SNP genotype and *Pde9a* expression, but since this did not correlate with phenotype, it was discounted as a candidate SNP. There were also SNP in non-essential splice sites in nine genes that may modify their exon usage (Supplementary Data S1: *AnnotatedFunctionalSNP.xls*).

### Correlation of haplotype assignments using 454 and Perlegen data

**Figure 4:**
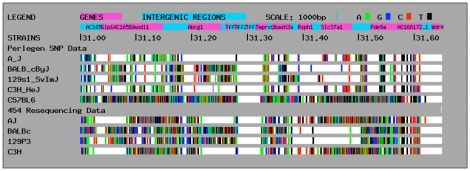
SNP plots of *Tir1* between 31 and 31.65 Mbp. The C57BL/6 row represents the reference allele for all loci that are polymorphic in either the Perlegen set or our 454 set. The SNP density is much greater in the 454 data set, in which haplotype blocks are clearly identifiable by eye. It appears that SNP are much better represented in the Perlegen data set in some regions than in others. Between 31.2 and 31.30 the two data sets are very similar with high density of SNP in BALB/c and 129 substrains in each dataset. However in the region between 31.1 and 31.20 the SNP in BALB/c and 129 are relatively much sparser in Perlegen than in the 454 data.

Jukes Cantor distances were calculated for each haplotype block in the *Tir1* region using the 454 and Perlegen datasets. A more detailed description of the analysis is presented in Supporting Text S1. Shared haplotypes had high positive predictive value and specificity for shared SNP alleles but low negative predictive value and sensitivity (Table S3b in Supporting Text S1), indicating that having shared haplotypes is a good indicator of shared SNP alleles but that the converse is not true. This means that assignments will be accurate where C57BL/6 has been assigned the same haplotype allele as susceptible strains but less accurate where C57BL/6 has been assigned to a different haplotype block allele from the susceptible strains. Therefore the data may be reliable way of excluding loci as candidate QTL regions but less accurate for including loci. The correlation between the distances calculated from the 454 and Perlegen SNP sets was modest (r = 0.63). The slope of the regression line was 0.67 reflecting the greater number of SNP in the 454 dataset. A high degree of scatter was observed in a plot of distances based on Perlegen and 454 data (Figure S3 in Supporting Text S1). The scatter suggests that SNP coverage is uneven in one or both datasets, and therefore increasing SNP density should increase the reliability of haplotype calls. Inspection of a plot of SNP coverage in the two data sets shows that the ratio of the number of SNP that were found in the two data sets varied substantially between haplotype blocks (Figure 4 and Figure S4 in Supporting Text S1).

Plots like those shown in Figures 4 and S3 can be obtained for any region of *Tir1* from our website [31]. Plots of SNP and haplotypes and tables of Jukes Cantor distances between alleles at each haplotype block based on Perlegen data can be obtained for any part of the mouse genome at the same site.

## Discussion

The survival time phenotype for mapping murine QTL associated with response to *T. congolense* infection was selected in the 1990's because the large variance between strains made it more likely that there would be QTL of large enough effect to be identifiable. This prediction proved correct [6], however survival is likely to have a remote and complex relationship with the underlying quantitative trait genes (QTG). Given that trypanosomiasis is a systemic blood stream infection and the remote relationship between survival and the underlying QTG it is almost impossible to prioritise candidate genes on the basis of known functions. We have previously measured parasitaemia, anaemia and fifteen clinical chemistry phenotypes, in inbred and congenic mice, in order to identify correlations between survival and other traits that might be more proximally related to gene function, however no such associations have been found [32]. Therefore in this study we have identified the allele carried at each QTL in an additional strain (C3H/HeJ), formally identified the physical boundaries of the QTL and enumerated CNV and functional SNP that fall within those boundaries.

### QTL mapping

The mapping studies showed that C3H/HeJ mice carry susceptible alleles at the *Tir1* and *Tir3* loci. No QTL were observed at the *Tir2* locus. The *Tir1* locus as defined by previous fine mapping studies is just proximal to the major histocompatibility complex (MHC) (Table 2), and the conversion of genetic distances to physical positions presented here shows that *Tir1* includes three classical class I MHC H2K genes. However previous studies have found no correlation between MHC haplotype and response to infection [4] consistent with the QTL gene not being a classical MHC molecule.

The mapping population was also screened for an association between *Tlr4* and survival; no association was found. This observation implies that the presence or absence of a functional *Tlr4* gene has no effect on survival, but does not preclude the pathway from *Tlr4* to *Nfkb* (nuclear factor kappa-B) from responding to infection. *Tlr4* could still participate in the regulation of anaemia and parasitaemia, which are not correlated with survival [11].

### Identification of physical boundaries of QTL

The two independent F6 and F12 mapping populations have reduced the 95% CI of the QTL to exceptionally small regions, particularly at *Tir1*, the QTL of largest effect, where the physical size of the 95% CI was 930 Kbp for the combined data from the A/J × C57BL/6 and BALB/c × C57BL/6 F6 crosses (Table 1). This was only twice the mean distance between markers at this locus (400 Kbp) and consequently the main limitation in identifying the boundaries of the QTL is in estimating the position of the peak.

### Identification of functional nsSNP

Resequencing of the QTL region on the Roche 454 platform at Liverpool to 15× coverage discovered 3,618 novel SNP loci that were deposited in dbSNP. Comparison with a resequencing project on the Illumina platform at the Wellcome Trust Sanger Institute to 22× coverage [13] showed 99.98% consistency in SNP calls even when no minimum coverage criterion was applied for calling a SNP. Both data sets contained large numbers of SNP called as heterozygotes with alternative allele frequencies between 25–80%. These loci from both data sets were associated with significantly higher sequence coverage in our data indicating that the majority were likely to be due to mapping artefacts probably caused by CNV. The 454 data contained only 71% of the SNP discovered by the higher coverage Illumina data but both methods discovered the same set of nsSNP. The 454 data discovered an additional 3% of SNP that were not in the Illumina data.

Utilising all SNP from the 454, Perlegen and Illumina data sets, three probably damaging nsSNP were identified in genes at the peak of the *Tir1* QTL that correlated perfectly with phenotype (Table 3). Two nsSNP were in *Pram1*; the *Pram1^537L/P^* polymorphism was scored as probably damaging by Polyphen. The *Pram1* ^103R/K^ polymorphism was classed as benign by Polyphen but lies within a proline rich domain (PRINTS: PR01217) that is involved in binding the “SH3 domain of hematopoietic progenitor kinase 1 (HPK-1)-interacting protein of 55 kDa (HIP-55),” which is known to stimulate the activity of HPK-1 and c-Jun N-terminal kinase (JNK)” [39]. *Pram1* is almost exclusively expressed in myeloid cells [36] and specifically in granulocytes in terminal stages of differentiation [40] where it is induced by retinoic acid. It was thought that *Pram1* might be a negative regulator of neutrophil differentiation since it is repressed in acute myeloid leukaemia. The deletion of *Pram1*, however, has no effect on neutrophil differentiation and maturation but does disrupt reactive oxygen intermediate production and degranulation by neutrophils [41]. This may affect the early, pro-inflammatory response to infection or downstream TNFa signalling, which has been shown to be differentially expressed in susceptible and resistant mice [42]. C57BL/6 appears to have the derived allele of *Pram1^537L/P^* since A/J, BALB/c and C3H/HeJ had the same allele as Hominidae and dogs. Since C57BL/6 tend to have a more inflammatory phenotype, it is possible that the polymorphisms lead to a gain of function with stronger binding to HIP55 leading to faster and more persistent ROI induction and a more inflammatory state.

The other probably damaging SNP at *Tir1* were *CR974462* and *Rgl2*. There is no annotation for CR974462. *Rgl2 (Rif)* is a small GTPase that is most highly expressed in macrophages and B cells and appears to be involved in *Ras* mediated signalling [43]. The *Rgl2* ^147H→Y^ polymorphism could affect the *Ras* pathway that plays a key role in leukocyte activation and is therefore a plausible candidate gene.

The Fas death domain-associated protein (*Daxx*) gene, which we have previously reported to contain a deletion of a single aspartate residue in susceptible mice [44], is also under the peak of *Tir1*. *Daxx* is within the MAPK pathway, which was found to respond to *T. congolense* infection in microarray data. However a new Polyphen analysis of the aspartate deletion in *Daxx* indicates that this polymorphism will be benign in effect. The aspartate deletion is within a run of 11 aspartate residues and a region where 35/41 residues are acidic [44]. Therefore this polymorphism is probably less significant than the probably damaging ones reported here.

Regulatory polymorphisms could also cause the phenotypic difference: one SNP (rs33223038) was identified in Ensembl as being in a regulatory region upstream of *Pde9a* but although this SNP correlated with differential expression it did not correlate with survival differences between susceptible and resistant mouse breeds. There were also SNP in non-essential splice sites in nine genes.

### Copy number variation at *Tir* QTL

CNV have previously been shown to be a major cause of quantative trait differences [10]. We used Agilent whole mouse genome aCGH arrays to identify CNV between C57BL/6 mice and A/J, BALB/c and 129P3 mice. The aCGH data highlighted a CNVR containing three genes close to the peak of the *Tir3c* QTL: *Cd244*; *Ly9* and *Itln1* (Figure 1A). A nearby gene *Cd48* had a probably damaging nsSNP. *Cd244*, *Cd48* and *Ly9* are important genes involved in the production and regulation of IFNg by NK and T cells. CD244 binds CD48 on lymphocytes and is involved in NK:NK cell and NK:T cell interactions leading to NK and T cell proliferation [45], which are important mechanisms in innate resistance to protozoan infection [46], [47].

Splenic expression of *Cd244* differed between strains with the resistant C57BL/6 mice having the lowest expression consistent with the low copy number of *Cd244* in C57BL/6. *Cd48* expression increased 16-fold in liver after infection with *T. congolense*, but this occurred in all strains tested (Figure 2). Since CD48 and CD244 directly interact, it is possible that the QTL is a consequence of the combined effect of the probably damaging nsSNP in *Cd48* and the CNV in *Cd244*. Differences in expression could not be seen in *Itlnb* or *Ly9*.

The large number of genes in *Tir3c* that had CNV, nsSNP or haplotypes that correlated with phenotype may make it difficult to identify the QTL gene at this locus. It is possible that the QTL is not a consequence of a single polymorphism but the combined effect of multiple polymorphisms in an extended haplotype. However the CNV at *Cd244* was the most substantial DNA polymorphism in the region making *Cd244* a strong candidate QTL gene. Inserting an additional copy of Cd244 into the C57BL/6 background, so that it had a similar gene dosage to the susceptible strains, could test the effect of this CNV on the response to infection.

### Haplotype block analysis

We have previously used this strategy to show a strong association between upstream haplotype differences and high confidence (p<0.005) differences in gene expression [19] and also short listed genes under QTL for differences in response to *Heligmosomoides bakeri* infection [20]. We reduced the number of candidate genes in this study by about 76% and 45% under hypotheses H1 and H2 from the 1193 genes that were under the 95% confidence intervals of the QTL. There were 283 genes where A/J, BALB/c and C3H/HeJ had the same haplotype different from C57BL/6 and 651 genes where C57BL/6 differed from the other three. The large number of genes that had haplotypes that correlated with phenotype is mainly because: 1) C3H/HeJ, A/J and BALB/c are more similar to each other than to any other strain based on analysis of 673 genome wide SNP in 55 strains [48]; 2) we used the stringent criterion that a gene was included if any haplotype block between the two neighbouring genes correlated with phenotype; 3) The high positive predictive power of the method means that whilst it is probably very reliable for excluding loci where susceptible strains share a haplotype block with the C57BL/6 resistant strains, it assigns too many haplotype blocks to different alleles.

Whilst there are large numbers of reported SNP for A/J, 129X1/SvJ and 129S1/SvImJ due to the Celera sequencing project [49] and for BALBc/ByJ and C3H/HeJ from the Perlegen project [15], relatively few SNP are publicly available for the 129P3 strain. The 454 resequencing of the *Tir1* region indicated that approximately 50% of the resequenced region could be excluded from the QTL if the allele carried by 129P3 mice at this locus was known. If a QTL was identified at *Tir1* in a 129P3 × C57BL/6 cross then the QTL gene could be assumed to be within the three blocks where 129P3 differed from C57BL/6. If no evidence of a QTL was found then these regions could be excluded from the QTL on the assumption that 129P3 carried the same allele as C57BL/6 at this locus. This analysis indicates that mapping QTL for response to infection in a 129P3 × C57BL/6 cross should significantly refine the list of candidate genes. The availability of this haplotype data makes it possible to make more rational choices about the selection of strains for mapping experiments. This strategy has been used before with a much more limited SNP set [50] but the corresponding online resource is no longer available.

Our objective was to identify the SNP that were most likely to have an impact on function. These were considered to be nsSNP that altered the physical properties of the protein as judged by Polyphen analysis, SNP in essential splice sites and CNV and regulatory SNP that correlated with changes in expression. It should be emphasised, however, that many types of SNP can underlie QTL, for example the QTL SNP at the *Idd5* locus appears to be a synonymous SNP that gives rise to a splice variant [51]. This SNP would not have been identified as a high priority by our pipeline. Furthermore, although we have substantially complete sequence coverage of the *Tir1* locus, at other loci we have used the Perlegen data, which is estimated to be about 45% complete [15]. Therefore although the candidate QTL SNP presented here are the most likely given the available data and annotation, both SNP data and annotation is incomplete and other candidates may be discovered in the future.

The correlation of Jukes-Cantor distances calculated from our 454 data and the published Perlegen dataset was only modest (*r* = 0.63). 32% of our 454 SNP loci were also in the Perlegen set, however the low correlation between the two sets shows that SNP discovery was uneven in one or both sets and inspection of the SNP distribution suggests that this was certainly the case in the Perlegen set. The uneven distribution of SNP discovery makes it much harder to undertake a consistent analysis across the genome using a single threshold for assigning alleles to haplotype blocks. However the high positive predictive value (Table S3 in Supporting Text S1) for identifying shared haplotypes suggests that this procedure should reliably exclude regions where C57BL/6 shares haplotypes with the susceptible strains. Nevertheless other more robust data types such as CNV and potentially functional SNP should still be surveyed in regions where haplotype does not correlate with phenotype. The more complete mouse resequencing projects that are currently underway should increase the predictive power of this approach substantially.

QTL involved with resistance to other parasitic diseases overlap with the *Tir* QTL, raising the possibility that polymorphisms discovered here may be involved in the response to other parasites. *Leishmania* resistance 1 (*Lmr1*) [52], *Plasmodium chabaudi* resistance QTL 3 (*Char3*) [53] and *Heligmosomoides bakeri* nematode resistance 2 (*Hbnr2*) [54] all overlap with *Tir1*. Similarly, the *Tir3c* QTL overlaps with a QTL for murine resistance to *Plasmodium berghei*-driven experimental cerebral malaria (*Berr1*) [55].

Thirteen genes around the peak of *Tir1* show conserved order and sequence homology to a ∼311 Kbp region of BTA7 (15,412,179∶15,723,462 bp) where there is a QTL in cattle that regulates the level of parasitaemia in cattle infections with *T. congolense* [2]. This region includes *Pram1*, which has a probably damaging mutation that correlates with phenotype in mice and was the most plausible candidate gene in *Tir1* and is therefore a candidate QTL gene in cattle as well. However since trypanotolerance QTL cover approximately 15% of the bovine genome it would be expected that at least one of the five murine QTL would coincide with a bovine QTL by chance (p = 0.56).

### Conclusions

By linking genes to haplotypes, we have reduced the number of candidate genes in *Tir1* to 43. Within these there were three genes with probably damaging nsSNP; CR974462.5, *Rgl2* and *Pram1*. CR974462.5 is an anonymous gene in which the effects are hard to predict. *Pram1* regulates oxidative stress in neutrophils and *Rgl2* is involved in *Ras* signalling which can regulate inflammation. *Pram1* is the closest to the peak of the QTL and has the best understood function making it the most attractive candidate at *Tir1* however *Rgl2* is also a plausible candidate. Probably damaging polymorphisms were identified in *Srp72* in *Tir2* and *Thsd7b* in *Tir3a* but little is known of their functions so it is hard to interpret these observations. *Ptprc (Cd45)* and *Soat1* in *Tir3b* had probably damaging polymorphisms in conserved nucleotides, CD45 is the common leukocyte antigen and has multiple roles in cytokine signaling and cell regulation making it plausible candidate. *Tir3c* has a CNVR encompassing *Cd244*, which is differentially expressed and has a haplotype that correlates with phenotype in the four strains tested. Since gene dosage is lower in C57BL/6 it will be possible to test this hypothesis by inserting an extra copy of the *Cd244* gene into the C57BL/6 background. Several other genes in *Tir3c* had haplotype and nsSNP that correlated with phenotype but none had such a distinct CNV and such strong differential expression.

By combining haplotype analysis, array-CGH, gene expression and next-generation DNA capture and sequencing, we have identified a small number of genetic polymorphisms that may be responsible for differences in response to *T. congolense* infection, demonstrating that this approach can systematically reduce the number of candidate genes under QTL to generate a short enough list of genes to test for function.

## Supporting Information

Data S1 AnnotatedFunctionalSNP.xls. A comprehensive annotation of publicly available SNP (NCBI build 37) across QTL regions including Polyphen annotation.
(1.97 MB XLS)

Data S2 GenesAndHaplotypes.xls. Haplotype block alleles across QTL regions
(1.02 MB XLS)

Data S3 SNP validation.xls. Comparison of resequencing of Tir1 region on the Illumina system at the Wellcome Trust Sanger Institute and on the 454 system at the University of Liverpool.
(0.04 MB XLS)

Text S1 Supporting Text referred to in the main text. Includes: additional methods on haplotype analysis and genotyping markers and primers; and additional SNP and CNV data.
(1.30 MB DOC)
