# Supplementary material for: A Comprehensive Genetic Analysis of Candidate Genes Regulating Response to Trypanosoma congolense Infection in Mice
Source: PLoS Negl Trop Dis. 2010 Nov 9;4(11):e880. doi: 10.1371/journal.pntd.0000880 (PMC2976683; doi:10.1371/journal.pntd.0000880)
Supplement: Text S1 — Supporting Text referred to in the main text. Includes: additional methods on haplotype analysis and genotyping markers and primers; and additional SNP and CNV data. (1.30 MB DOC) [file pntd.0000880.s004.doc]

**Supplementary Data**

**A comprehensive genetic analysis of candidate genes regulating response to *Trypanosoma congolense* infection in mice**

Authors:

Ian Goodhead^1^; Alan Archibald^5^; Peris Amwayi^2^; Andy Brass^3,4^; John Gibson^2,£^; Neil Hall^1^; Margaret Hughes^1^; Moses Limo^6^; Fuad Iraqi^2$^; Steve Kemp^1,2^ and Harry Noyes^1*^

Institutions:

1. Centre for Genomic Research. School of Biological Sciences, University of Liverpool. L69 7ZB. UK
2. International Livestock Research Institute, Box 30709, Nairobi 00100, Kenya
3. Faculty of Life Sciences, University of Manchester, Michael Smith Building, Oxford Road, Manchester, M13 9PT, UK
4. School of Computer Science, Kilburn Building, University of Manchester, Oxford Road, Manchester, M13 9PL, UK
5. The Roslin Institute, University of Edinburgh, Roslin, EH25 9PS, Scotland, UK
6. Egerton University, Njoro, Nakuru, Kenya

* Corresponding Author

^£^ Present address: The Centre For Genetic Analysis and Applications, C.J. Hawkins Homestead, University of New England, Armidale, NSW 2351, Australia.

^$^ Present address: Department of Clinical Microbiology and Immunology, Sackler Faculty of Medicine, Tel Aviv University, Ramat Aviv, Tel Aviv 69978, Israel.

Author Contact Information

IG – ian.goodhead@liv.ac.uk

AA - alan.archibald@roslin.ed.ac.uk

PA - pwamwayi@icipe.org

JG - jgibson5@une.edu.au

SK - kempsj@liverpool.ac.uk

AB - andy.brass@manchester.ac.uk

FI - fuadi@post.tau.ac.il

ML - mklimoh@yahoo.com

NH - neilhall@liverpool.ac.uk

MH - tm0s00a0@liverpool.ac.uk

Corresponding Author Contact Information

Harry Noyes

School of Biological Sciences

University of Liverpool

Crown Street

Liverpool

L69 7ZB

+44 (0)151 795 4512

harry@liv.ac.uk

**Identification of QTL in C3H/HeJ mice**


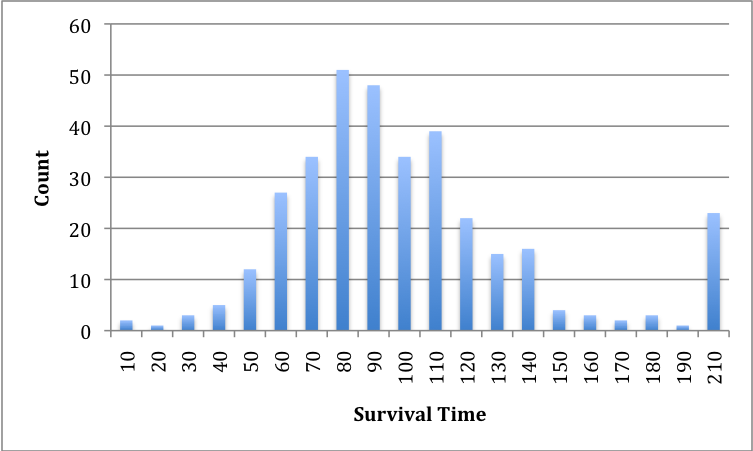


**Figure S1** Distribution of survival times of C3H/HeJ × C57BL/6 F2 mice after infection with *T. congolense* IL1180. Labels on the X-axis indicate the start of each interval; hence the interval labeled 10 includes animals that died between days 10-19. 94 animals that had extreme survival times ≤62 days and >140 days were selected for genotyping using the markers shown in Table S1. The peak at 210 days is for mice that were surviving at the end point of the experiment. They were included in the genotyping and their survival time was recorded as 141 days.

**Table S1**: List of markers assayed for resistant (C57BL/6) or susceptible (C3H/HeJ) alleles in *T. congolense*-infected mice. 345 F2 C3H/HeJ × C57BL/6 mice were phenotyped for survival time after infection with *T. congolense* strain IL1180. 94 animals that had extreme survival times were selected for genotyping using these markers. Mean survival for mice at each marker for each given genotype is shown.

| **Marker** | **Type of Marker** | **Chromosome** | **Mean Survival  Homozygous C3H/HeJ (days)** | **Mean Survival  Homozygous C57BL/6 (days)** | **Mean Survival Heterozygous (days)** | **χ^2^ Significant difference in survival between homozygous genotypes** |
| --- | --- | --- | --- | --- | --- | --- |
| D17mit117 | Microsatellite | 17 | 57.50 | 123.45 | 100.55 | 5.84E-07 |
| D17mit155 | Microsatellite | 17 | 97.50 | 112.49 | 105.18 | 3.00E-01 |
| D17Mit184 | Microsatellite | 17 | 72.58 | 118.74 | 102.65 | 5.36E-04 |
| D17Mit68 | Microsatellite | 17 | 56.09 | 121.74 | 99.94 | 3.94E-07 |
| D17mit93 | Microsatellite | 17 | 90.08 | 116.87 | 100.05 | 6.17E-02 |
| D1mit155 | Microsatellite | 1 | 72.42 | 112.30 | 108.69 | 9.63E-04 |
| D1mit215 | Microsatellite | 1 | 99.61 | 118.35 | 103.63 | 1.72E-01 |
| D1mit425 | Microsatellite | 1 | 86.55 | 121.72 | 104.72 | 1.51E-02 |
| D1mit94 | Microsatellite | 1 | 93.89 | 117.70 | 99.95 | 1.02E-01 |
| D5mit169 | Microsatellite | 5 | 96.56 | 105.10 | 109.06 | 3.72E-01 |
| D5mit201 | Microsatellite | 5 | 106.36 | 78.00 | 107.52 | 7.02E-03 |
| D5mit255 | Microsatellite | 5 | 103.71 | 133.50 | 108.14 | 6.74E-03 |
| D5mit81 | Microsatellite | 5 | 103.63 | 140.00 | 104.07 | 8.35E-04 |
| rs46746692 | SNP | 5 | 101.97 | 113.20 | 100.33 | 4.15E-01 |
| rs46742668 | SNP | 5 | 107.55 | 99.00 | 106.89 | 4.98E-01 |
| rs47415520 | SNP | 5 | 0.00 | 107.90 | 0.00 | N/A |
| rs31694652 | SNP | 5 | 109.21 | 115.30 | 98.76 | 3.21E-01 |
| rs13465576 | SNP | 17 | 62.42 | 115.93 | 108.36 | 1.51E-05 |
| D4mit178 | Microsatellite | 4 | 105.65 | 110.83 | 103.42 | 6.18E-01 |
| rs3023006 | SNP | 4 | 104.47 | 111.14 | 104.04 | 5.88E-01 |

**Table S2:** Primer sequences used for SNP genotyping

| **ID** | **Forward Primer (5’-3’)** | **Reverse Primer (5’-3’)** |
| --- | --- | --- |
| **rs13465576** | GGCTGCTTTCTGAGTCCAAG | GAACAGGGAAAATGGCTGAA |
| **rs46746692** | GATCTGGGGCAGCTCTTGA | CATTTTACAGCAGGGTATTATGG |
| **rs46742668** | ACGGTTAGCAGAGGAGGATG | TGTTGTTGTTGTGTGTTTTGTTTT |
| **rs47415520** | CATCCTGATTGGTCATCTCC | TTTAGGGAGGCAAAATTCCA |
| **rs31694652** | GACCTGAGGTGTCTTTTTCTTCA | CCTCAGCTGGTTTCAGTACCA |
| **rs3023006** | GGACTGGGTGAGAAATGAGC | GAAACTGCCATGTTTGAGCA |

**Allocation of strains to haplotypes**

Haplotypes that are shared between two mouse strains are segments of the genome that are assumed to have descended form a common ancestor. If we assume that the reason that two mouse strains have different phenotypes is because they have inherited a certain region of the genome from different ancestors, then by identifying the regions where two mouse strains with different phenotypes have different ancestral haplotypes, then we can obtain a shorter list of likely candidate genes. It is of course possible that the difference in phenotype is a consequence of a recent mutation that does not correlate with haplotype, particularly when considering only two strains. So whilst haplotype difference can be a useful guide for prioritising candidate genes it is not a necessarily the case that a QTL gene will be in a region where haplotypes differ.

The boundaries of haplotypes have been published [3], but the allocation of strains to haplotypes is not available on a genome wide scale. The single nucleotide polymorphism (SNP) alleles for over 40 mouse strains at each of over 8 million SNP loci were found by Perlegen Corporation under a contract from NIH [3]. In order to decide whether strains shared a haplotype the sequences of haplotype blocks were used to estimate genetic distances between strains and then an algorithm was applied to allocate strains to the same or different haplotypes.

Haplotype boundaries were downloaded from http://mouse.perlegen.com/mouse/
download.html [3]. Strains were allocated to haplotypes for each haplotype block using a local Perl script that extracted all alleles from the Perlegen dataset within a haplotype block, substituted the polymorphic positions into the C57BL/6 reference sequence and submitted the aligned sequences to the Jukes-Cantor algorithm in DNADIST in PHYLIP to calculate genetic distances between each pair of strains [4]. Strains were allocated to the same haplotype if the genetic distance was ≤0.00005. C57BL/6 was used as the reference strain for block allocation and assigned to haplotype one, succeeding strains were allocated to the same haplotype block as another strain they shared a haplotype with or, if they did not share the haplotype with any other strain, to the next available haplotype number. Haplotype assignments for each Haplotype block used in the present study are shown in the Supplementary Data S2 - *GenesAndHaplotypes.xls*. Haplotype blocks for any region of the genome can be obtained from: http://www.genomics.liv.ac.uk/tryps/GeneExpressionViewer/
MouseSNP-HaplotypeViewer.html. Users can set any reasonable threshold at which to allocate blocks to haplotypes and download tables of block allocations and Jukes-Cantor distances between alleles. All scripts are available from the authors on request.

The distribution of distances between strains was examined in R in order to determine the most appropriate threshold value to use to allocate strain to the same or different haplotypes (Figure S2). The raw distances were not normally distributed but the ln transformed distances approximated to a normal distribution. The 5% quantile of the distribution was at exp(-9.9) = 0.00005. This value was used as a threshold at which to assign strains to a haplotype. If the Jukes Cantor distance between two strains for a haplotype block was less than 0.00005 the two strains were assigned to the same haplotype block. Other wise they were assigned to different haplotype blocks.


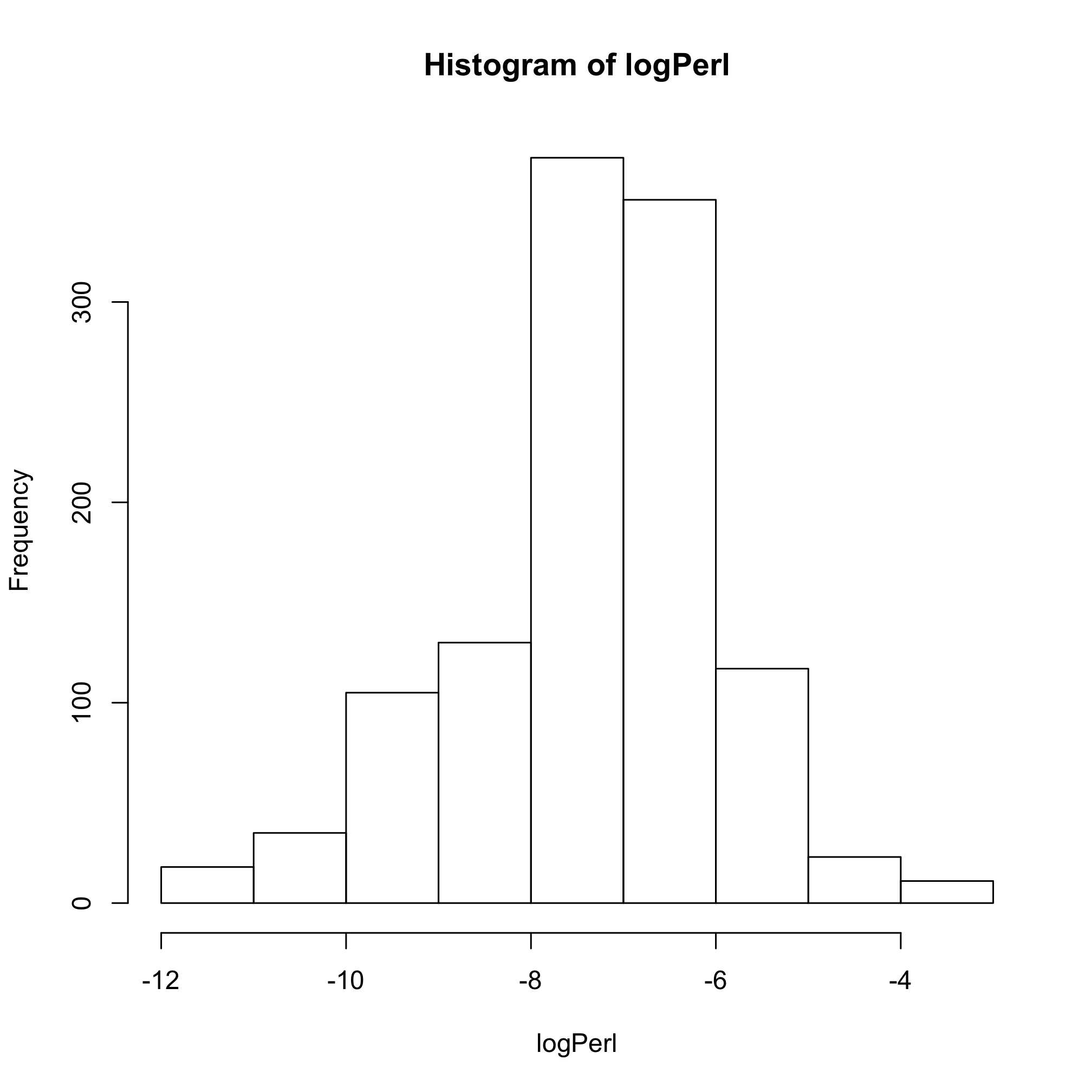


Ln distance between strains

**Figure S2**. Histogram of ln transformed distances between strains for the *Tir1* region obtained using SNP in the Perlegen set. Distance was calculated using the Jukes-Cantor method in DNADIST in Phylip as described above. The fifth percentile of the distribution was at exp(-9.9) = 0.00005.

**Comparing genetic distances based on the Perlegen data set and the dataset obtained by resequencing the *Tir1* region.**

Since many novel SNP and alleles were discovered in the *Tir1* region by 454 resequencing the genetic distances between haplotype alleles would be expected to be larger when using the 454 data set. The correlation between distances determined from the two SNP sets is an indication of relative evenness of coverage of the two sets. Figure S3 shows a scatter plot of distances calculated using the 454 data against the distances calculated using the Perlegen data. The correlation between the distances calculated from the two SNP sets was modest (r = 0.63). The slope of the regression line was 0.67 reflecting the greater number of SNP in the 454 dataset. The high degree of scatter suggests that SNP coverage is uneven in one or both datasets, and therefore increasing SNP density should increase the reliability of haplotype calls. The power of haplotypes to predict SNP allele status was estimated.

**Table S3: A** Counts of SNP that are consistent with haplotype allocation for the Perlegen and 454 data sets for comparisons between C57BL/6 and A/J and between C57BL/6 and BALB/c. For any comparison SNP may have the same allele (match) or different alleles (mismatch). Haplotypes may also have the same allele (match) or different (mismatch). **B** The haplotypes had high positive predictive value and sensitivity for SNP allele but low sensitivity and negative predictive value. This means that they accurately assigned shared haplotype alleles but was less accurate for assigning strains to different haplotype block alleles.

**A**

| Perlegen data  (Whole genome) | | SNP | |
| --- | --- | --- | --- |
|  |  | Match | Mismatch |
| Haplotypes | Match | 7,248,641 | 25,970 |
|  | Mismatch | 5,591,688 | 1,960,979 |
|  |  |  |  |
|  |  |  |  |
| 454 data (Tir1 region) | | SNP | |
|  |  | Match | Mismatch |
| Haplotypes | Match | 4,365 | 431 |
|  | Mismatch | 8,317 | 13,977 |

B

|  | Perlegen Data | 454 Data |
| --- | --- | --- |
| Positive Predictive value | 1.00 | 0.91 |
| Negative Predictive value | 0.74 | 0.37 |
| Sensitivity | 0.56 | 0.34 |
| Specificity | 0.99 | 0.97 |

**Figure S3**. Scatter plot of Jukes Cantor distances obtained using the Perlegen data and our 454 resequencing data. The natural log of the Jukes Cantor distance was calculated for each pair of strains for each haplotype block in the *Tir1* region using our 454 data and the Perlegen data set. The distances obtained using each SNP dataset were then plotted against each other. r = 0.63; *y* = 0.6687*x* - 2.0259

In order to make a public resource for comparing the two datasets we have established a website at http://www.genomics.liv.ac.uk/tryps/resources.html which displays graphical views of the SNP in any part of the genome and plots of haplotype assignments based on those SNP (Figure S4).

Haplotype blocks of SNP stand out clearly in the 454 data in the example shown in Figure S4. The haplotype blocks and their boundaries are much less obvious in the Perlegen data. However it appears that SNP are much better represented in some regions than in others. Between 31.2 and 31.30 the two data sets are very similar with high density of SNP in BALB/c and 129 substrains in each dataset. However in the region between 31.1 and 31.20 the SNP in BALB/c and 129 are relatively much sparser in Perlegen than in the 454 data. This is consistent with the high degree of scatter in Figure S3, which suggested that the two SNP sets did not both capture a consistent proportion of the underlying variation across the whole genome. Relative to 454, the Perlegen data set contained a higher proportion of SNP in some regions than in others. In the absence of a perfect reference set it is not possible to say whether the 454 data also captures variable proportions of the SNP in different regions.


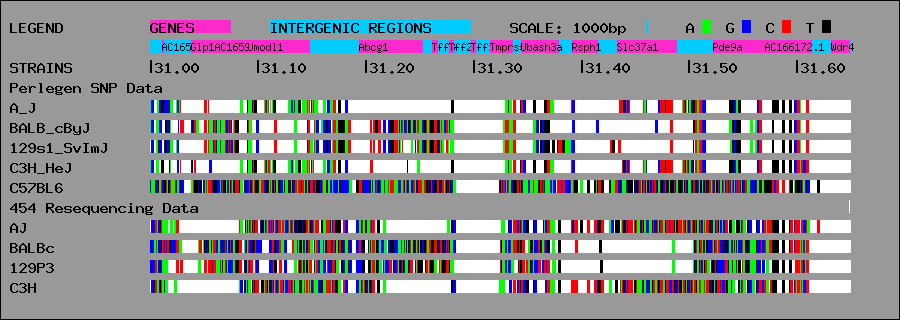


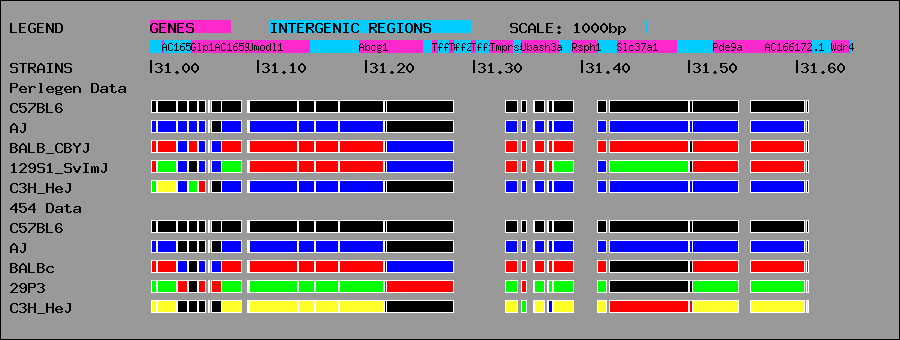


**Figure S4**. SNP and haplotype plots of *Tir1* between 31 and 31.65Mb. The upper panel shows SNP in the Perlegen and 454 datasets. The C57BL/6 row represents the reference allele for all loci that are polymorphic in either the Perlegen set or our 454 set. The SNP density is clearly much greater in the 454 data set in which haplotype blocks are clearly identifiable by eye. The lower panels show haplotype block alleles assigned on the basis of genetic distances. Pairs of strains that are more than a threshold genetic distance apart are allocated to different alleles. The threshold used in this example was 0.00005 as described above, but any reasonable value can be set by the user. At this threshold there were more alleles of many haplotype blocks when using the 454 data than when using the Perlegen data. This is presumably a reflection of the greater SNP density in the 454 dataset.

**Supplementary SNP Data**

**Table S4:** Summary statistics for 454 GS-FLX Titanium sequencing and mapping to the C57BL/6 reference.

Filtered SNP are those SNP remaining after filtering to remove SNP: within a 13bp window of homopolymeric tracts; outside capture probe regions; with sequence coverage  ≥8X, 7 of which must match the alternative allele. Novel genotypes are SNP either not previously characterised, or disagree with previous genotypes (dbSNP128).

|  | **A/J** | **BALB/cJ** | **C3H/HeJ** | **Common Loci**  **(3 strains)** | **129P3/J** | **Common Loci**  **(4 strains)** |
| --- | --- | --- | --- | --- | --- | --- |
| **Total SNP** | 19950 | 19136 | 12890 |  | 5616 |  |
| **Filtered SNP** | 7969 | 7435 | 6046 | 1588 | 2160 | 466 |
| **Novel Genotype** | 1615 | 7327 | 4207 | 150 | 2160 | 36 |
| **% Novel genotypes at Novel Loci** | (94%) | (25%) | (30%) | (9%) | (19%) | (8%) |

| ***Difference Position (mmu17 / bp)*** | ***Reference Allele*** | ***Alternative Allele*** | ***Data Type*** | ***GeneID*** | ***Region of Gene*** |
| --- | --- | --- | --- | --- | --- |
| 30728075 | C | T |  | Glo1 |  |
| 30728075 | C | T |  | Glo1 |  |
| 30728075 | C | T |  | Glo1 |  |
| 30728075 | C | T |  | Glo1 |  |
| 31,011,574 | G | A | H3K4me3:ESHyb | AC165951.3-1 | Upstream |
| 31,194,080 | T | G | H3K4me3:ES | Abcg1 | Upstream |
| 31,194,238 | G | C | H3K4me3:ES | Abcg1 | Upstream |
| 31,522,166 | A | G | DNase1:ES | Pde9a | Upstream |
| 31979094 | GAC | - |  | Snf1lk |  |
| 31979094 | GAC | - |  | Snf1lk |  |
| 31979094 | GAC | - |  | Snf1lk |  |
| 31979094 | GAC | - |  | Snf1lk |  |
| 31,979,290 | T | C | DNase1:ES | Snf1lk | Upstream |
| 35,669,957 | C | A | DNase1:ES | Psors1c2 | Upstream |
| 35,771,262 | T | C | DNase1:ES | Dpcr1 | Upstream |
| 35,772,620 | T | C | H3K4me3:ES | Dpcr1 | Upstream |
| 35,790,848 | T | G | DNase1:ES | Vars2 | Upstream |
| 35,790,864 | T | G | DNase1:ES | Vars2 | Upstream |
| 35,835,340 | T | C | H3K4me3:ES | U6 | Upstream |
|  |  |  |  |  |  |
| 31,046,212 | G | A | DNase1:ES | Glp1r | Within |
| 31,991,185 | A | G | DNase1:ES | Snf1lk | Within |
| 33,276,221 | T | C | DNase1:ES | Morc2b | Within |
| 35,692,027 | T | C | H3K4me3:ESHyb | Cdsn | Within |
| 35,692,042 | T | C | H3K4me3:ESHyb | Cdsn | Within |
| 35,692,339 | T | C | H3K4me3:ESHyb | Cdsn | Within |
| 35,705,160 | T | C | DNase1:ES | 2300002M23Rik | Within |
| 35,773,710 | T | C | DNase1:ES | Dpcr1 | Within |
| 35,819,115 | A | T | DNase1:ES | Ddr1 | Within |
| 35,819,115 | A | T | DNase1:ES | Ddr1 | Within |
| 36,326,297 | A | G | H3K27me3:ES | H2-T3 | Within |

**Table S5**: List of SNPs in chromosome 17 matching regions of accessible chromatin according to the Ensembl murine functional genomics database. SNPs matched identically in all four strains of susceptible mice after mapping against the resistant C57BL/6 reference with at least 50% of the reads agreeing at a given consensus position. Differences are either within 2.5Kbp ‘upstream’ in possible promoter binding regions; or are in accessible chromatin regions ‘within’ the coding regions of genes that may be associated with transcription factor binding sites.

Mouse regulatory build data from Ensembl Mouse Build 37:

<http://www.ensembl.org/info/docs/funcgen/index.html>;

DNAse1:ES - DNase1 hypersensitivity sites

[1].

H3K4me:ESHyb; H3K27me3:ES - Histone methylation possibly associated with promoter regions [2].

## SNP Validation

This comparison was exclusively of SNP within capture regions and focuses on two classes of polymorphism:

1. Homozygous single base polymorphisms (excluding indels) in which a single allele is observed in the sequenced strain;
2. Heterozygous single base polymorphisms in which the sequenced strain has two alleles at a specific position.

**Table S6** In 454 data Homozygous is defined as Coverage >= 1 and alternate allele frequency (AAF) > 80% (mean coverage was 12 and AAF 98%) and Heterozygous is Coverage > 8 and alternate allele frequency > 25-80%. Heterozygous alleles cannot be detected at low coverage values.

|  | 454 | Illumina |
| --- | --- | --- |
| Homozygous | 37,617 | 49,858 |
| Heterozygous | 7,136 | 9,377 |

Since the strains sequenced are assumed to almost completely inbred it is possible that the substantial numbers of heterozygous SNP are derived from duplicated regions. If this were the case, then it would be expected that the SNP that were heterozygous in the Illumina data would be in regions of higher coverage in the 454 data. The mean coverage in the 454 data of positions matching homozygous Illumina SNP was 12 and of those matching heterozygous SNP was significantly higher at 19 (p > 10^-99^), therefore it seems likely that a substantial proportion of the heterozygous SNP are artefacts of the assembly and have been disregarded.

**Concordance of SNP common to 454 and Sanger data sets**

The number of common SNP depended on the criteria used to call a SNP. Concordance was measured with coverage greater than each number between 1 and 8 and AAF greater than 0 or 80.

**Table S7:** Concordance of allele calls between 454 and Illumina data, where there was data at a given position in both data sets, was almost perfect. Only 5 out of nearly 37,000 calls (0.014%) were discordant even with no coverage or AAF filters. Applying these filters made a negligible difference to the number of discordant alleles although it reduced the number of common positions because fewer 454 positions passed these criteria.

| **Minimum Coverage** | **AAF** | **Identical Alleles** | **Different Alleles** |
| --- | --- | --- | --- |
| **1** | 0 | 36784 | 5 |
| **3** | 0 | 33700 | 5 |
| **5** | 0 | 30404 | 4 |
| **7** | 0 | 27073 | 4 |
| **1** | 80 | 34472 | 3 |
| **3** | 80 | 31561 | 3 |
| **5** | 80 | 28804 | 2 |
| **7** | 80 | 25625 | 2 |

**Missing data in Sanger and 454 sets**

The overlap between the Sanger and 454 sets depended on the criterion used for calling a SNP in the 454 data. At a minimum coverage of 8X, 53% of SNP in Sanger were also in 454 but at a minimum coverage of 1X, 71% of SNP in Sanger data were also in 454.


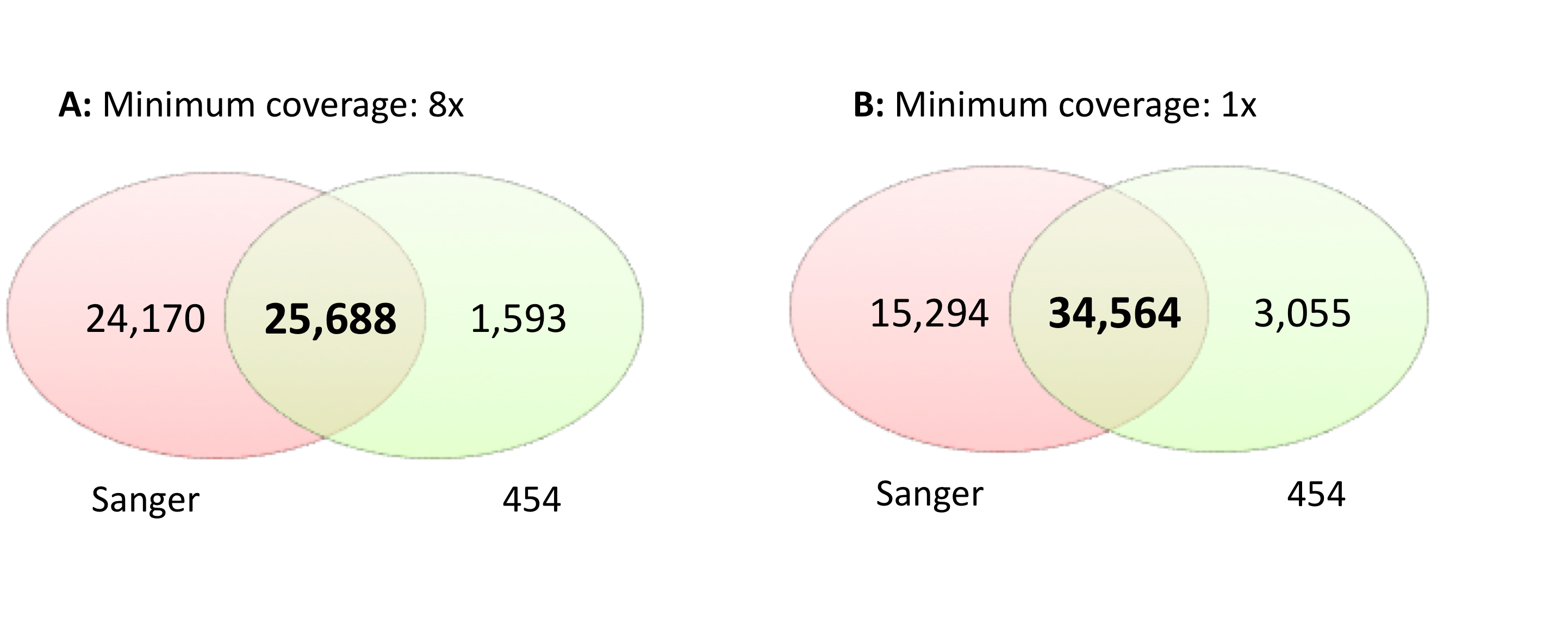


**Figure S5** Venn diagrams showing overlap in SNP between Sanger and 454 SNP. The extent of overlap is dependent on the minimum coverage used for calling a SNP. All figures are based on a minimum minor allele frequency of 80%.

**Supplementary CNV Data**

**Table S8:** A list of significant CNVR in C57BL/6 (resistant) relative to A/J, BALB/c and 129P3 (susceptible) mice. Negative scores indicate deletions in C57BL/6 and positive scores amplifications, respectively. Genes predicted to be involved with chemosensory pathways have been removed due to having large local variations in copy number [5]. Aberrations were grouped into copy number variant regions (CNVR) using the Agilent CGH Analytics Software “Common Aberration test” (Overlap: 0.9; p<0.01) using the ADM-2 algorithm (Threshold: 6.0) with Centralization (Threshold: 6; Bin Size: 1) and Fuzzy Zero [6]. CNVR positions were converted to NCBI37 using Liftover (http://genome.ucsc.edu/cgi-bin/hgLiftOver). Expression data for liver (L), spleen (S) and kidney (K) tissue was not available for all genes.

| **Chromosome (Tir region)** | **CNV Start (bp)** | **CNVR End (bp)** | **# CNV Probes** | **Common Aberration  P value** | **Score** | **CNV Location (Relative to gene)** | **Gene Symbol** | **Expression difference between breeds? (Tissue)** | **Expression responds to infection?** |
| --- | --- | --- | --- | --- | --- | --- | --- | --- | --- |
| **17 (Tir1)^§^** | 30,586,088 | 31,060,347 | 68 | <1.4x10^-237**^ |  | Gene within CNVR | *Glo1* | **Yes (L)** | **Yes** |
|  |  |  |  |  |  | Gene within CNVR | *Dnahc8* | **Yes (S)** | **No** |
|  |  |  |  |  |  | Gene within CNVR | *Gpr1* | **No** | **No** |
|  |  |  |  |  |  | Gene within CNVR | *AC165951.3-1* | n/a | |
|  |  |  |  |  |  | Gene within CNVR | *AC125544.4* | n/a | |
|  |  |  |  |  |  | Border | *Btbd9* | **Yes** | **No** |
| **1 (Tir3c)** | 173,441,746 | 173,499,029 | 11 | 0.00029 | -1104 | Border | *Cd244* | **Yes (L,S)** | **Yes** |
|  |  |  |  |  |  | Gene within CNVR | *AC083892.19-1* | n/a | |
|  |  |  |  |  |  | Gene within CNVR | *Itlnb* | n/a | |
| **3** | 142,269,450 | 142,286,796 | 4 | 0.002 | -15.84 | Border | *Gbp1* | **Yes** | **Yes** |
| **4** | 62,157,766 | 62,182,695 | 8 | 0.005 | -58.13 | Gene within CNVR | *Alad* | **Yes (L,S)** | **Yes (L)** |
|  |  |  |  |  |  | Gene within CNVR | *Hdhd3* | n/a | |
| **4** | 111,725,815 | 113,560,896 | 106 | 0.004 | 11.7 | Gene within CNVR | *9530098N22Rik* | n/a | |
|  |  |  |  |  |  | Gene within CNVR | *A430090E18Rik* | n/a | |
|  |  |  |  |  |  | Gene within CNVR | *A030013N09Rik* | n/a | |
|  |  |  |  |  |  | Gene within CNVR | *Skint6* | n/a | |
| **6** | 129,600,838 | 129,618,743 | 4 | 0.014 | 5.83 | Border | *Klrc1* | **No** | **No** |
|  |  |  |  |  |  | Border | *Klrc2* | **No** | **No** |
| **6** | 129,690,146 | 129,733,410 | 8 | 0.025 |  | Gene Within CNVR | *Gm156* | n/a | |
|  |  |  |  |  |  | Gene Within CNVR | *Klri2* | n/a | |
| **6** | 129,936,848 | 130,172,513 | 26 | 0.03 | 9.57 | Complex | *Klra* | **Yes (S) ^¶^** | **Yes ^¶^** |
| **7** | 111,427,300 | 111,514,865 | 7 | 0.008 | 16.53 | Border | *Trim34* | **No** | **Yes** |
| **7** | 111,644,545 | 111,694,781 | 7 | 0.003 | 12.36 | Border | *AI451617* | n/a | |
|  |  |  |  |  |  | Gene within CNVR | *EG625321* | n/a | |
| **14** | 69877096 | 70083115 | 32 | 0.002 | 32 | Border | *Loxl2* | **No** | **No** |
|  |  |  |  |  |  | Border | *Slc25a37* | n/a | |
|  |  |  |  |  |  | Gene Within CNVR | *D930020E02Rik* | n/a | |
|  |  |  |  |  |  | Gene Within CNVR | *Entpd4* | n/a | |
| **X** | 166,413,387 | 166,422,251 | 3 | 6.81x10^-5^ | 85.69 | CNVR within gene | *Mid1* | **Yes(S)** | **No** |
|  |  |  |  |  |  | CNVR within gene | *G530011O06Rik* | n/a | |

**§: Amplification only occurs in 129/J and A/J mice.**

****: P value too low for software to determine precise p value for A/J (reported as ‘zero’). P value quoted for *Glo1* is for 129/J only.**

**#: CNVR varies between breeds and only reported by algorithm in BALBc and A/J mice, although smaller amplification can be seen in 129/J (data not shown). P value and score reported is for CNVR based on A/J and BALB/c only.**

**¶: *Klra2*, *Klra3* and *Klra7* show reduced expression in C57BL/6 mice compared to susceptible breeds. All except *Klra5* and *Klra6* show differences in expression through infection. Expression data only available for *Klra 1-3; 5-9; 12,13,16* and *17*.**

Accompanying Supplementary Data files

Supplementary Data S1 - *AnnotatedFunctionalSNP.xls*

Supplementary Data S2 - *GenesAndHaplotypes.xls*

Supplementary References

1. Crawford GE, Holt IE, Whittle J, Webb BD, Tai D, et al. (2006) Genome-wide mapping of DNase hypersensitive sites using massively parallel signature sequencing (MPSS). Genome Res 16: 123-131.

2. Barski A, Cuddapah S, Cui K, Roh TY, Schones DE, et al. (2007) High-resolution profiling of histone methylations in the human genome. Cell 129: 823-837.

3. Frazer KA, Eskin E, Kang HM, Bogue MA, Hinds DA, et al. (2007) A sequence-based variation map of 8.27 million SNPs in inbred mouse strains. Nature.

4. Felsenstein J (2005) PHYLIP (Phylogeny Inference Package) version 3.6.

5. Nozawa M, Nei M (2008) Genomic drift and copy number variation of chemosensory receptor genes in humans and mice. Cytogenet Genome Res 123: 263-269.

6. Lipson D, Aumann Y, Ben-Dor A, Linial N, Yakhini Z (2006) Efficient calculation of interval scores for DNA copy number data analysis. J Comput Biol 13: 215-228.
